# Supplementary material for: Sociosexual behavior requires both activating and repressive roles of Tfap2e/AP-2ε in vomeronasal sensory neurons
Source: eLife. 2022 Sep 16;11:e77259. doi: 10.7554/eLife.77259 (PMC9525060; doi:10.7554/eLife.77259)
Supplement: Supplementary file 1. [file elife-77259-supp1.docx]

| **Supplementary File 1** | | |  |  |  |
| --- | --- | --- | --- | --- | --- |
| **Differentially expressed genes in mature apical and basal VSNs** | | | | | |
| **Apically Enriched Genes** | |  |  |  |  |
| **Gene Name** | **P. Value** | **Average Log Fold Change** | **Mature Basal %** | **Mature Apical %** | **Adjusted P.Value** |
| Nsg1 | 2.19E-13 | -1.9461937 | 0.87 | 0.991 | 4.77E-09 |
| Rtp1 | 3.99E-13 | -1.7210806 | 0.13 | 0.972 | 8.69E-09 |
| Gnai2 | 1.21E-12 | -3.2758293 | 0.261 | 0.963 | 2.63E-08 |
| Gng13 | 1.44E-12 | -2.4998775 | 0.565 | 0.981 | 3.14E-08 |
| Meis2 | 1.46E-12 | -2.3994924 | 0.087 | 0.944 | 3.18E-08 |
| Cystm1 | 2.60E-12 | -1.1223905 | 0.913 | 1 | 5.66E-08 |
| Gng2 | 4.70E-12 | -1.8780287 | 0.696 | 0.963 | 1.02E-07 |
| Fam241a | 2.15E-11 | -1.3441602 | 0.304 | 0.925 | 4.68E-07 |
| Pcdh7 | 5.79E-11 | -1.1606574 | 0.043 | 0.869 | 1.26E-06 |
| Plekhb1 | 3.29E-10 | -0.8073116 | 0.739 | 0.991 | 7.16E-06 |
| Ckb | 5.70E-10 | -0.942904 | 0.957 | 1 | 1.24E-05 |
| Jakmip1 | 2.13E-09 | -0.7864416 | 0.13 | 0.841 | 4.65E-05 |
| Nrp2 | 2.48E-09 | -0.9997662 | 0.565 | 0.925 | 5.41E-05 |
| G630016G05Rik | 3.62E-09 | -0.8518453 | 0 | 0.766 | 7.89E-05 |
| Gramd1c | 6.12E-09 | -0.8132581 | 0.304 | 0.869 | 1.33E-04 |
| Car2 | 1.29E-08 | -0.871219 | 0.783 | 0.963 | 2.82E-04 |
| Socs2 | 1.40E-08 | -0.8762327 | 0.913 | 0.981 | 3.04E-04 |
| Aig1 | 1.33E-07 | -0.6442775 | 0.478 | 0.869 | 2.91E-03 |
| Sgpl1 | 1.38E-07 | -0.750675 | 0.652 | 0.953 | 3.01E-03 |
| Ppp1r1a | 1.62E-07 | -0.6387545 | 0.87 | 0.953 | 3.53E-03 |
| Nfix | 1.63E-07 | -0.6919687 | 0.087 | 0.71 | 3.55E-03 |
| Rtp2 | 2.67E-07 | -0.6124008 | 0.783 | 0.925 | 5.81E-03 |
| Eml2 | 3.24E-07 | -0.4522206 | 0 | 0.645 | 7.05E-03 |
| Spef2 | 3.76E-07 | -0.6257323 | 0.522 | 0.907 | 8.19E-03 |
| Cartpt | 6.02E-07 | -0.504793 | 0.957 | 0.963 | 1.31E-02 |
| Esd | 6.05E-07 | -0.5783619 | 0.174 | 0.748 | 1.32E-02 |
| Tspan1 | 7.99E-07 | -0.6642304 | 0.304 | 0.785 | 1.74E-02 |
| S100a13 | 1.18E-06 | -0.6578302 | 0.217 | 0.72 | 2.58E-02 |
| Zdhhc3 | 1.22E-06 | -0.6494611 | 0.609 | 0.879 | 2.65E-02 |
| Bag1 | 1.60E-06 | -0.4936463 | 0.87 | 0.972 | 3.48E-02 |
| Exosc7 | 1.70E-06 | -0.4935659 | 0.522 | 0.841 | 3.71E-02 |
| Prxl2a | 1.95E-06 | -0.6048927 | 0.957 | 1 | 4.24E-02 |
|  |  |  |  |  |  |
| **Basally Enriched Genes** | |  |  |  |  |
| **Gene Name** | **P. Value** | **Average Log Fold Change** | **Mature Basal %** | **Mature Apical %** | **Adjusted P.Value** |
| Tfap2e | 2.71E-23 | 1.01282475 | 0.87 | 0.019 | 5.90E-19 |
| Cnpy1 | 1.23E-22 | 2.05509001 | 1 | 0.093 | 2.69E-18 |
| Robo2 | 3.77E-22 | 0.90330448 | 0.957 | 0.056 | 8.20E-18 |
| Tafa1 | 4.72E-21 | 0.96728089 | 0.826 | 0.028 | 1.03E-16 |
| Vmn2r1 | 2.11E-19 | 1.2793116 | 0.783 | 0.028 | 4.59E-15 |
| Sphkap | 6.49E-15 | 0.28031925 | 0.522 | 0 | 1.41E-10 |
| Gnao1 | 6.12E-14 | 1.42314493 | 0.957 | 0.327 | 1.33E-09 |
| Apmap | 1.17E-13 | 1.58179511 | 1 | 0.916 | 2.55E-09 |
| Gm36028 | 1.19E-13 | 2.53665489 | 1 | 0.682 | 2.58E-09 |
| Krt18 | 2.60E-13 | 1.6051759 | 1 | 0.673 | 5.67E-09 |
| Calr4 | 6.57E-13 | 1.39565197 | 1 | 0.916 | 1.43E-08 |
| Krt8 | 1.51E-12 | 1.68676872 | 0.957 | 0.551 | 3.28E-08 |
| Fam3c | 1.65E-12 | 1.07729387 | 1 | 0.664 | 3.60E-08 |
| Dio3 | 1.68E-12 | 0.40528977 | 0.478 | 0.009 | 3.65E-08 |
| Agpat5 | 1.79E-12 | 1.08439932 | 1 | 0.514 | 3.89E-08 |
| Sdf2l1 | 5.07E-12 | 1.50705224 | 1 | 0.879 | 1.10E-07 |
| Pdia3 | 6.16E-12 | 0.86129121 | 1 | 1 | 1.34E-07 |
| Itm2b | 7.96E-12 | 1.18425959 | 1 | 1 | 1.73E-07 |
| Manf | 1.07E-11 | 1.37611844 | 1 | 0.953 | 2.33E-07 |
| Cfap300 | 2.16E-11 | 0.77700954 | 0.957 | 0.421 | 4.71E-07 |
| Creld2 | 2.17E-11 | 1.00229149 | 0.957 | 0.523 | 4.73E-07 |
| Shisa8 | 2.58E-11 | 0.36615576 | 0.391 | 0 | 5.63E-07 |
| Fkbp2 | 2.90E-11 | 0.72341813 | 1 | 0.907 | 6.32E-07 |
| Hspa5 | 5.22E-11 | 1.40010137 | 1 | 0.981 | 1.14E-06 |
| Dnajc3 | 6.37E-11 | 1.1592286 | 0.957 | 0.729 | 1.39E-06 |
| Dio3os | 7.37E-11 | 0.49488572 | 0.565 | 0.047 | 1.60E-06 |
| Pdia6 | 1.07E-10 | 1.14235131 | 1 | 0.888 | 2.32E-06 |
| Hsp90b1 | 2.05E-10 | 0.85553036 | 1 | 0.991 | 4.47E-06 |
| Dut | 2.84E-10 | 0.76430257 | 1 | 0.86 | 6.19E-06 |
| Mfge8 | 3.07E-10 | 1.07748862 | 0.957 | 0.794 | 6.68E-06 |
| Vmn2r2 | 4.22E-10 | 1.35916407 | 0.391 | 0.009 | 9.19E-06 |
| Dpysl3 | 1.30E-09 | 1.17193306 | 0.957 | 0.757 | 2.84E-05 |
| E330013P04Rik | 1.41E-09 | 0.35778211 | 0.609 | 0.084 | 3.08E-05 |
| Mt3 | 1.88E-09 | 0.57279227 | 0.739 | 0.215 | 4.09E-05 |
| Tubb3 | 3.51E-09 | 0.64687805 | 1 | 0.963 | 7.64E-05 |
| Prdx2 | 3.78E-09 | 0.72277812 | 1 | 0.953 | 8.23E-05 |
| Osbpl9 | 5.01E-09 | 0.83250073 | 0.913 | 0.813 | 1.09E-04 |
| Fbxo17 | 5.56E-09 | 0.50343797 | 0.652 | 0.15 | 1.21E-04 |
| Stbd1 | 6.55E-09 | 1.09297275 | 1 | 0.963 | 1.43E-04 |
| Slc35b1 | 9.68E-09 | 0.68626655 | 0.957 | 0.738 | 2.11E-04 |
| Rd3 | 1.35E-08 | 0.5858953 | 1 | 0.972 | 2.93E-04 |
| Gchfr | 1.35E-08 | 0.32772543 | 0.522 | 0.065 | 2.94E-04 |
| Ppib | 1.55E-08 | 0.6188014 | 1 | 0.972 | 3.38E-04 |
| Selenof | 1.55E-08 | 0.51120029 | 1 | 0.981 | 3.38E-04 |
| Plxdc2 | 1.59E-08 | 0.2695123 | 0.435 | 0.037 | 3.45E-04 |
| Ugp2 | 1.63E-08 | 0.90939627 | 0.957 | 0.794 | 3.56E-04 |
| Tmed3 | 2.62E-08 | 0.50570554 | 1 | 0.916 | 5.71E-04 |
| B2m | 3.78E-08 | 1.26181829 | 0.913 | 0.542 | 8.23E-04 |
| Akr1b3 | 6.06E-08 | 0.97983293 | 0.87 | 0.523 | 1.32E-03 |
| Optn | 7.24E-08 | 0.31830823 | 0.478 | 0.065 | 1.58E-03 |
| Golim4 | 8.68E-08 | 0.52488666 | 0.913 | 0.551 | 1.89E-03 |
| Selenom | 1.04E-07 | 0.49334844 | 1 | 1 | 2.26E-03 |
| Tppp3 | 1.17E-07 | 0.89025439 | 0.652 | 0.168 | 2.55E-03 |
| Racgap1 | 1.58E-07 | 0.61403494 | 0.913 | 0.561 | 3.45E-03 |
| Rhou | 1.72E-07 | 0.39239137 | 0.609 | 0.15 | 3.74E-03 |
| Dnajb11 | 1.76E-07 | 0.70097289 | 0.957 | 0.944 | 3.84E-03 |
| Mfap3l | 2.28E-07 | 0.28167932 | 0.565 | 0.103 | 4.96E-03 |
| Kctd1 | 2.38E-07 | 0.88812556 | 0.913 | 0.542 | 5.17E-03 |
| Traf3ip3 | 3.03E-07 | 0.73231367 | 0.391 | 0.037 | 6.61E-03 |
| Galnt18 | 3.24E-07 | 0.55665364 | 0.826 | 0.411 | 7.05E-03 |
| Calb2 | 3.73E-07 | 0.43308595 | 1 | 1 | 8.13E-03 |
| Cdkn1a | 3.82E-07 | 0.5373044 | 0.957 | 0.907 | 8.33E-03 |
| Spcs3 | 4.63E-07 | 0.55754661 | 0.957 | 0.832 | 1.01E-02 |
| Dusp26 | 4.98E-07 | 0.54873277 | 0.957 | 0.822 | 1.08E-02 |
| Bri3bp | 6.03E-07 | 0.36483009 | 0.522 | 0.112 | 1.31E-02 |
| Vmn2r53 | 1.01E-06 | 2.68138046 | 0.217 | 0 | 2.21E-02 |
| Spcs2 | 1.09E-06 | 0.59703272 | 0.957 | 0.944 | 2.37E-02 |
| Carhsp1 | 1.32E-06 | 0.38531423 | 0.522 | 0.112 | 2.87E-02 |
| Dclk1 | 2.00E-06 | 0.39895571 | 0.826 | 0.327 | 4.35E-02 |
| Guk1 | 2.05E-06 | 0.5985497 | 0.913 | 0.897 | 4.45E-02 |
| B230217C12Rik | 2.24E-06 | 0.424191 | 1 | 0.813 | 4.87E-02 |
