# Supplementary material for: Sociosexual behavior requires both activating and repressive roles of Tfap2e/AP-2ε in vomeronasal sensory neurons
Source: eLife. 2022 Sep 16;11:e77259. doi: 10.7554/eLife.77259 (PMC9525060; doi:10.7554/eLife.77259)
Supplement: Supplementary file 2. [file elife-77259-supp2.docx]

| **Supplementary File 2** | | |  |  |  |
| --- | --- | --- | --- | --- | --- |
| **Significantly dysregulated genes when Tfap2e/AP-2ε is ectopically expressed in apical VSNs** | | | | | |
| **Significantly Down-Regulated Genes** | | |  |  |  |
| **Gene Name** | **P. Value** | **Average Log Fold Change** | **Mature ­­­­­­­**  **OMPCre^+/-^/R26AP2ε^+/-^ Apical %** | **Mature**  **OMPCre^+/-^Apical %** | **Adjusted P.Value** |
| Nsg1 | 1.05E-19 | -1.026049429 | 0.978 | 0.991 | 2.28E-15 |
| Ckb | 3.16E-13 | -0.832807392 | 0.957 | 1 | 6.89E-09 |
| Plekhb1 | 1.02E-12 | -0.66365877 | 0.804 | 0.991 | 2.23E-08 |
| Calr | 1.99E-12 | -0.848017913 | 0.326 | 0.879 | 4.33E-08 |
| Gng13 | 3.92E-11 | -0.851799068 | 0.978 | 0.981 | 8.55E-07 |
| Prdx1 | 6.92E-11 | -0.670948424 | 0.674 | 0.925 | 1.51E-06 |
| Ftl1-ps1 | 5.23E-10 | -1.01755918 | 0.5 | 0.822 | 1.14E-05 |
| Calm2 | 7.50E-09 | -0.359436172 | 1 | 1 | 1.63E-04 |
| Rps4x | 9.27E-09 | -0.462870937 | 0.978 | 1 | 2.02E-04 |
| Chgb | 5.21E-08 | -0.509353353 | 0.63 | 0.907 | 1.13E-03 |
| Obp2a | 5.51E-08 | -0.803452485 | 0.5 | 0.832 | 1.20E-03 |
| Esd | 1.16E-07 | -0.447142323 | 0.391 | 0.748 | 2.54E-03 |
| Eml2 | 1.21E-07 | -0.290177152 | 0.152 | 0.645 | 2.63E-03 |
| Lcn3 | 3.31E-07 | -0.704072872 | 0.413 | 0.804 | 7.22E-03 |
| Nqo1 | 4.44E-07 | -1.080448365 | 0.239 | 0.636 | 9.68E-03 |
| Ppp1r1a | 6.98E-07 | -0.395511356 | 0.935 | 0.953 | 1.52E-02 |
| Id4 | 9.43E-07 | -0.48810942 | 0.283 | 0.673 | 2.05E-02 |
| Tspan1 | 1.12E-06 | -0.469458509 | 0.543 | 0.785 | 2.45E-02 |
| Cpe | 1.20E-06 | -0.310695303 | 1 | 1 | 2.61E-02 |
| Gt(ROSA)26Sor | 1.32E-06 | -0.45231242 | 0.717 | 0.832 | 2.88E-02 |
| Tstd1 | 1.35E-06 | -0.387425488 | 0.935 | 0.963 | 2.94E-02 |
|  |  |  |  |  |  |
| **Significantly Up-Regulated Genes** | | |  |  |  |
| **Gene Name** | **P. Value** | **Average Log Fold Change** | **Mature ­­­­­­­**  **OMPCre^+/-^/R26AP2ε^+/-^ Apical %** | **Mature**  **OMPCre^+/-^Apical %** | **Adjusted P.Value** |
| Abca7 | 2.79E-10 | 0.467733988 | 0.848 | 0.393 | 6.07E-06 |
| Apmap | 4.12E-08 | 0.499754357 | 1 | 0.916 | 0.000898417 |
| Calb2 | 2.04E-08 | 0.32207374 | 1 | 1 | 0.00044526 |
| Calr4 | 6.89E-11 | 0.580533012 | 0.935 | 0.916 | 1.50177E-06 |
| Ccdc136 | 1.17E-08 | 0.433981247 | 0.696 | 0.252 | 0.000254306 |
| Cdkn1a | 2.88E-07 | 0.455507857 | 1 | 0.907 | 0.006262153 |
| Cst6 | 3.50E-14 | 1.099290617 | 0.935 | 0.794 | 7.62225E-10 |
| Dbi | 3.62E-12 | 1.062519896 | 0.957 | 0.813 | 7.8747E-08 |
| Dio3os | 1.34E-15 | 0.383916442 | 0.652 | 0.047 | 2.92422E-11 |
| Dlx4 | 8.01E-09 | 0.438918572 | 0.783 | 0.327 | 0.000174394 |
| Dnajc3 | 5.15E-07 | 0.423663136 | 0.913 | 0.729 | 0.011213757 |
| Dusp15 | 1.60E-08 | 0.288331862 | 0.587 | 0.14 | 0.00034899 |
| Dync1i1 | 3.35E-08 | 0.495088228 | 0.848 | 0.533 | 0.000729768 |
| Enpp1 | 1.92E-06 | 0.36206672 | 0.891 | 0.551 | 0.041912856 |
| Fam3c | 3.92E-07 | 0.381794339 | 0.913 | 0.664 | 0.008538247 |
| Fbxo17 | 1.06E-17 | 0.668034836 | 0.804 | 0.15 | 2.31481E-13 |
| Fmnl2 | 1.47E-06 | 0.294102212 | 0.717 | 0.346 | 0.031962548 |
| Gm36028 | 1.51E-15 | 1.565013376 | 0.935 | 0.682 | 3.27889E-11 |
| Gnao1 | 5.65E-07 | 0.344705173 | 0.783 | 0.327 | 0.012315414 |
| Golim4 | 1.06E-06 | 0.393270819 | 0.804 | 0.551 | 0.023038974 |
| Itpr1 | 1.54E-12 | 0.42801754 | 0.696 | 0.131 | 3.35489E-08 |
| Jak1 | 1.57E-07 | 0.423690646 | 0.978 | 0.972 | 0.003418952 |
| Kctd1 | 7.64E-16 | 0.85428255 | 0.978 | 0.542 | 1.66452E-11 |
| Kctd17 | 5.75E-08 | 0.383015359 | 0.957 | 0.907 | 0.001251818 |
| Khdrbs1 | 9.29E-07 | 0.388803088 | 0.935 | 0.692 | 0.020230178 |
| Krt18 | 2.14E-06 | 0.448568282 | 0.935 | 0.673 | 0.046678066 |
| Lrrc58 | 5.13E-09 | 0.336180339 | 0.717 | 0.243 | 0.00011176 |
| Mab21l2 | 3.38E-11 | 0.342240813 | 0.37 | 0 | 7.3587E-07 |
| Mapk8ip2 | 1.52E-07 | 0.38275929 | 1 | 0.963 | 0.003310007 |
| Mfap3l | 5.77E-11 | 0.330339336 | 0.609 | 0.103 | 1.25577E-06 |
| Mfge8 | 7.50E-08 | 0.517438515 | 0.913 | 0.794 | 0.001634157 |
| Mgat5 | 3.40E-07 | 0.328523537 | 0.87 | 0.486 | 0.007398101 |
| Mt3 | 2.31E-12 | 0.486337274 | 0.761 | 0.215 | 5.03186E-08 |
| Osbpl9 | 3.00E-09 | 0.615805448 | 0.957 | 0.813 | 6.544E-05 |
| Pir | 6.37E-09 | 0.572417884 | 0.848 | 0.486 | 0.000138796 |
| Ptprd | 4.71E-07 | 0.333394508 | 0.522 | 0.14 | 0.010266165 |
| Racgap1 | 1.11E-06 | 0.405891754 | 0.87 | 0.561 | 0.024189968 |
| Rims3 | 2.59E-07 | 0.430808566 | 1 | 0.916 | 0.005638623 |
| Rmdn3 | 2.84E-08 | 0.443857099 | 0.783 | 0.458 | 0.000618452 |
| Rpl11 | 2.14E-07 | 0.287691748 | 0.978 | 0.981 | 0.004668027 |
| Smchd1 | 6.17E-13 | 1.30491047 | 0.935 | 0.598 | 1.34339E-08 |
| Spats2 | 3.38E-12 | 0.613696552 | 0.957 | 0.832 | 7.36795E-08 |
| Stbd1 | 1.62E-08 | 0.724116114 | 0.978 | 0.963 | 0.000353362 |
| Suox | 9.51E-08 | 0.433632116 | 0.739 | 0.364 | 0.002071784 |
| Tafa1 | 1.26E-13 | 0.39251169 | 0.522 | 0.028 | 2.74227E-09 |
| Tfap2e | 7.33E-30 | 2.020231614 | 0.935 | 0.019 | 1.597E-25 |
| Tsnax | 2.00E-08 | 0.574771495 | 1 | 1 | 0.000435139 |
